# Exploring Preterm Birth as a Polymicrobial Disease: An Overview of the Uterine Microbiome

**DOI:** 10.3389/fimmu.2014.00595

**Published:** 2014-11-27

**Authors:** Matthew S. Payne, Sara Bayatibojakhi

**Affiliations:** ^1^School of Women’s and Infants’ Health, The University of Western Australia, Perth, WA, Australia

**Keywords:** preterm birth, bacteria, virus, fungi, yeast, infection, amniotic fluid, placenta

## Abstract

Infection is a leading cause of preterm birth (PTB). A focus of many studies over the past decade has been to characterize microorganisms present in the uterine cavity and document any association with negative pregnancy outcome. A range of techniques have been used to achieve this, including microbiological culture and targeted polymerase chain reaction assays, and more recently, microbiome-level analyses involving either conserved, phylogenetically informative genes such as the bacterial 16S rRNA gene or whole shotgun metagenomic sequencing. These studies have contributed vast amounts of data toward characterization of the uterine microbiome, specifically that present in the amniotic fluid, fetal membranes, and placenta. However, an overwhelming emphasis has been placed on the bacterial microbiome, with far less data produced on the viral and fungal/yeast microbiomes. With numerous studies now referring to PTB as a polymicrobial condition, there is the need to investigate the role of viruses and fungi/yeasts in more detail and in particular, look for associations between colonization with these microorganisms and bacteria in the same samples. Although the major pathway by which microorganisms are believed to colonize the uterine cavity is vertical ascension from the vagina, numerous studies are now emerging suggesting hematogenous transfer of oral microbiota to the uterine cavity. Evidence of this has been produced in mouse models and although DNA-based evidence in humans appears convincing in some aspects, use of methodologies that only detect viable cells as opposed to lysed cells and extracellular DNA are needed to clarify this. Such techniques as RNA analyses and viability polymerase chain reaction are likely to play key roles in the clinical translation of future microbiome-based data, particularly in confined environments such as the uterus, as detection of viable cells plays a key role in diagnosis and treatment of infection.

## Introduction

It has been well established that infection is a leading cause of preterm birth (PTB) and is highly associated with the deliveries that occur at the earliest gestations (1, 2). Although several theories have been proposed outlining the establishment of intra-uterine infections, the most widely accepted is that microorganisms residing in the vagina vertically migrate through the cervix, colonize the fetal membranes and then subsequently, the amniotic fluid (AF), placenta, and fetus (1). More recently, particularly in relation to bacteria colonizing the placenta, evidence has been presented suggesting hematogenous spread of organisms from the mother to the amniotic cavity (3–5).

For many years, studies have attempted to document these organisms, first using conventional microbiological culture and in the past two decades using a combination of culture, organism-specific polymerase chain reaction (PCR) assays and in the case of bacteria, 16S rDNA phylogenetics using a range of approaches including molecular cloning, denaturing gradient gel electrophoresis (DGGE), and terminal restriction fragment length polymorphism (TRFLP) analyses. These studies have contributed a wealth of information to our knowledge of what microorganisms colonize the uterine cavity. Recent advances in molecular biology that have seen the widespread use of next-generation sequencing (NGS) platforms for both amplicon and whole genome sequencing (WGS) have further enhanced our knowledge of these organisms, particularly those that represent very small proportions of a given microbial community.

This review aims to provide an overview of the total microbiome of the uterine cavity and discuss associations between specific organisms and negative pregnancy outcome. Some recent literature constantly use the term “microbiome” to describe bacterial microbiota, when in actual fact this term is really all encompassing and refers to microorganisms in general, including bacteria, viruses, yeasts, and fungi. As such, we will provide an overview of the bacterial, viral, and yeast/fungal microbiomes of the two main uterine compartments examined to date, the AF and placenta. In addition, we will discuss some of the limitations associated with current uterine microbiome data in terms of our ability to translate findings into clinical practice, as well as examining the potential implications of viewing PTB as a polymicrobial condition.

## Amniotic Fluid Microbiome

Since the discovery of bacteria in the AF of cesarean section pregnancies by Harris and Brown in 1927 (6), the previously held belief that the fetus developed in a sterile environment has been challenged. Now, in the present day era of advanced molecular microbiological methodologies, we are well aware that numerous microbial organisms colonize the uterine environment (7, 8), many of which have been causally linked to PTB.

### Bacteria

Without a doubt, of all components of the uterine microbiome the greatest amount of data available relates to bacteria. Recent reviews by DiGiulio (7) and Mendz et al. (8) have provided a thorough overview of the major bacterial genera and species associated with AF colonization in cases of PTB. We have provided a summary of these and more recent bacterial microbiome studies in Table 1.

**Table 1:** **Overview of the major molecular^a^ bacterial microbiome-based analyses of the uterine cavity**.

Authors/year	Sample	Subjects	Organisms detected	Major findings
Jalava et al. (10)	AF^b^	20 cases of PPROM^b^; 16 controls (term)	**PPROM:** *Ureaplasma urealyticum*, *Haemophilus influenzae*, *Streptococcus oralis*, and *Fusobacterium* sp.	25% of samples were 16S rDNA positive for bacterial DNA. *U. urealyticum* was detected on two occasions
			**Controls:** no bacteria were detected	
Markenson et al. (11)	AF	54 cases of PTL^b^	No sequencing of amplicons was conducted	55.5% of samples were 16S rDNA positive
Hitti et al. (12)	AF	69 cases of PTL with intact membranes	Group B Streptococci, *Enterococcus* sp., *Escherichia coli*, *Klebsiella pneumoniae*, *Mycoplasma hominis*, *Gardnerella vaginalis*, *Fusobacterium nucleatum*, *Bacteroides ureolyticus*, *Prevotella oulora*, *Clostridium* sp., and *Peptostreptococcus asaccharolyticus*	Bacteria were identified from 36% of culture-negative samples using PCR
Gardella et al. (13)	AF	69 cases of PTL	*Leptotrichia sanguinegens*, *F. nucleatum*, *U. urealyticum*, and an uncultured oral bacterium	16S rDNA PCR and sequencing are promising techniques to identify bacteria from culture-negative samples
DiGiulio et al. (14)	AF	166 cases of PTL	*M. hominis*, *Ureaplasma* sp., *Streptococcus agalactiae*, *Lactobacillus* sp., *Prevotella* sp., *F. nucleatum*, *Streptococcus mitis*, *uncultivated Bacteroidetes bacterium*, *Delftia acidovorans*, *Neisseria cinerea*, *Sneathia sanguinegens*, *Leptotrichia amnionii*, and an uncultured bacterium	17 women had positive results for bacterial 16S rDNA
Han et al. (15)	AF	46 cases of PTB^b^; 16 controls (term)	**PTB:** *L. sanguinegens*, *S. sanguinegens*, *B. ureolyticus*, *Citrobacter koseri*, *Bacteroides fragilis*, *F. nucleatum*, *Prevotella bivia*, *Shigella* sp., Clostridiales bacterium, *Bergeyella* sp., *Ureaplasma parvum*, *S. agalactiae*, *L. amnionii*, *M. hominis*, and *Peptostreptococcus* sp.	45% of AF samples were positive for bacterial 16S rDNA. The most abundant 16S rDNA sequence detected was *F. nucleatum* (33.3%)
			**Controls:** no bacteria were detected	
Jones et al. (16)	FM^b^ and PLAC^b^	26 cases of PPROM; 19 cases of PTL with intact membranes; 8 cases of indicated PTL; 21 controls (term)	**CS term:** no bacteria were detected	PTL samples showed a higher prevalence and diversity of bacteria. Blood monocyte counts in PTL and PPROM groups that were positive for 16S rDNA were indicative of suppressed immunity. 30, 43, and 19% of samples were positive using broad-range 16S rDNA PCR, species-specific real-time PCR and a combination of both methods, respectively. 60% of PTL samples had multibacterial infection. The most commonly detected organisms were *U. parvum* followed by *Fusobacterium* sp.
			**V term:** *U. parvum*, *Lactobacillus crispatus*, *Fusobacterium* sp., *Pantoea* sp., and *Eubacterium rectale*	
			**CS indicated PTB:** *Fusobacterium* sp.	
			**CS PTL with PROM:** *U. parvum*, *S. mitis* group, *Fusobacterium* sp., *Veillonella parvula*, *H. influenzae*, and *U. urealyticum*	
			**V PTL with PROM:** *U. parvum*, *Fusobacterium* sp., *S. agalactiae*, *M. hominis*, *Atopobium vaginae*, *L. crispatus*, *E. coli*, *Peptoniphilus lacrimalis*, *Corynebacterium amycolatum*, and *U. urealyticum*	
			**V PTL with intact membranes:** *U. parvum*, *Fusobacterium* sp., *S. agalactiae*, *S. mitis* group, *L. crispatus*, *H. influenzae*, *Oribacterium sinus*, *Veillonella* sp., *Peptostreptococcus* sp., *Enterobacter aerogenes*, *Corynebacterium aerogenes*, *G. vaginalis*, *Finegoldia magna*, *Peptoniphilus asaccharolyticus*, *Streptococcus anginosus*, and *B. ureolyticus*	
DiGiulio et al. (17)	AF	52 cases of SGA^b^ neonates	*Staphylococcus epidermidis* and *S. agalactiae*	Two bacteria positive samples were identified
DiGiulio et al. (18)	AF	62 cases of preeclampsia	*Lactobacillus iners*, *S. anginosus*, *Corynebacterium tuberculostearicum*, *Ureaplasma* sp., and *Sneathia/Leptotrichia* sp.	8% of samples were positive for bacterial DNA. *Ureaplasma* sp. *and Sneathia/Leptotrichia* were the most frequently detected bacteria in cases of MIAC.
DiGiulio et al. (19)	AF	204 cases of PPROM	*Prevotella oris*, *Prevotella copri*, *Bacteroides* sp., *B. fragilis*, *Myroides* sp., *F. nucleatum*, *Fusobacterium* sp., *Leptotrichia* sp., *S. sanguinegens*, *L. amnionii*, *Dialister* sp., *Streptococcus* sp., *Streptococcus salivarius*, *S. agalactiae*, *Enterococcus faecalis*, *Listeria monocytogenes*, *Staphylococcus equorum*, *Staphylococcus pettenkoferi*, *Staphylococcus* sp., *Lactobacillus delbrueckii*, *Lactobacillus gasseri*, *Coprobacillus* sp., *Peptostreptococcus* sp., *Filifactor alocis*, *Clostridiaceae* sp., *Clostridium hiranonis*, *Brachybacterium* sp., *Rothia dentocariosa*, *Bifidobacterium longum*, *Bifidobacterium pseudolongum*, *G. vaginalis*, *A. vaginae*, *Ureaplasma* sp., *M. hominis*, *Neisseria subflava*, *Kingella denitrificans*, *H. influenzae*, *Haemophilus haemoglobinophilus*, *Haemophilus parainfluenzae*, *Campylobacter* sp., and an uncultured bacterium	A 45% prevalence of MIAC in the study group was recorded. 44 bacterial species were identified using PCR. The most common organism detected was *Ureaplasma* sp.
Marconi et al. (20)	AF	20 cases of PTL and 20 controls (term)	**PTL:** *B. fragilis*, *P. bivia*, *L. amnionii*, *M. hominis*, and *U. urealyticum*	40% of PTL and 5% of control cases were positive for MIAC
			**Controls:** *M. hominis*	
Wang et al. (21)	AF and CB^b^	36 cases of PTB, IAI^b^ or EONS^b^, and 8 controls (term)	**AF bacteria:** *E. coli*, *S. agalactiae*, *M. hominis*, *P. bivia*, *Lachnospiraceae* sp., *U. parvum*, *Peptoniphilus harei*, *S. sanguinegens*, *S. pneumoniae*, *B. ureolyticus*, *Bergeyella* sp., *S. mitis*, *L. monocytogenes*, *H. influenzae*, and *F. nucleatum*	31 and 18 bacterial species were identified in AF and CB, respectively. *E. coli* and *F. nucleatum* were the most frequently detected bacteria
			**CB bacteria:** *E. coli*, *S. agalactiae*, *F. nucleatum*, *M. hominis*, *U. parvum*, *Bergeyella* sp., and *S. sanguinegens*	
			**Controls:** no bacteria were detected	
Romero et al. (22)	AF	142 cases of PTL	*U. parvum*, *F. nucleatum*, *G. vaginalis*, *M. hominis*, *U. urealyticum*, *Acinetobacter junii*, *Sneathia* sp., *Pseudomonas* sp., *Aeromonas caviae*, *Moraxella osloensis*, *Staphylococcus aureus*, *Acidovorax* sp., *Lactobacillus* sp., *Pantoea dispersa*, and *Streptococcus* sp.	MIAC was present in 21% of cases. The most commonly detected bacteria was *U. parvum*
Combs et al. (9)	AF	305 cases of PTL	*B. ureolyticus*, *S. sanguinegens*, *F. nucleatum*, *G. vaginalis*, *H. influenzae*, *U. urealyticum*, *U. parvum*, *S. agalactiae*, *Bacteroides hemolyticus*, *L. monocytogenes*, *Bergeyella zoohelecum*, *Bergeyella* sp. *Staphylococcus hemolyticus*, and *L. amnionii*	MIAC was detected in 10% of AF samples
Aagaard et al. (3)	PLAC	320 pregnancies (preterm/term)	*E. coli*, *Escherichia* sp., *Prevotella tannerae*, *Bacteroides* sp., *Streptomyces avermitilis*, *Propionibacterium acnes*, *Rhodococcus erythropolis*, *Neisseria polysaccharea*, *Neisseria lactamica*, *Fusobacterium* sp., *Streptosporangium* sp., *Roseovarius* sp., *Rhodococcus* sp., *Paenibacillus* sp., *Klebsiella* sp., *Burkholderia* sp., and *Anaeromyxobacter* sp.	*E. coli* was the most commonly detected bacteria in the placenta. The placental microbiome is unique and harbors a variety of non-pathogenic commensal bacterial species. It is most closely related to the oral microbiome

^a^Whole genome shotgun sequencing, broad-range16S rDNA, or a combination of broad-range 16S rDNA and targeted PCR assays;

*^b^AF, amniotic fluid; FM, fetal membranes; PLAC, placenta; CB, cord blood; PTL, preterm labor; V, vaginal delivery; CS, Cesarean section; PPROM, preterm premature rupture of membranes; PTB, preterm birth; SGA, small gestational age; IAI, intraamniotic infection; EONS, early onset neonatal sepsis*.

The most recent study documenting AF infection was conducted by Combs et al. (9), and examined 305 cases of women in spontaneous preterm labor with intact fetal membranes using a combination of enrichment culture and 16S rDNA cloning. Of the 305 cases, they reported the presence of bacteria in 30 AF samples, 26 of which they attributed to infection based upon elevated levels of interleukin-6 (IL-6) (>11.2 ng/mL) and 4 of which were deemed “colonizers” due to levels of IL-6 <2.6 ng/mL. The most common organisms identified were *Ureaplasma urealyticum* (11 cases), *Fusobacterium nucleatum* (5 cases), *Bacteroides ureolyticus* (4 cases), *Sneathia sanguinegens* (4 cases), *Ureaplasma parvum* (4 cases), and *Streptococcus agalactiae* (3 cases).

Interestingly, Combs et al. (9) also reported numerous cases of culture-positive, PCR-negative detection (65% positive by both, 16% by culture only, and 19% by PCR only), and concluded that the techniques are complementary and that neither can be relied upon 100% for detection of AF infection. A similar result was shown by DiGiulio et al. (14) where they reported six culture-positive samples that were negative by PCR and nine PCR-positive samples that were negative by culture. A potentially important consideration that may explain this disparity exists in the very small volume of AF used for DNA extraction in the DiGiulio et al. (14) study (0.2 mL). The amount of AF used by Combs et al. (9) in DNA extractions is not provided and neither study details the volumes of sample used in culture analyses, although DiGiulio et al. (14) state they centrifuged samples for culture and resuspended them in 1 mL of supernatant, so we assume the original volume was >1 mL in these cases. Considering the generally low titers of bacteria found in AF samples, for molecular detection it would appear to be beneficial to use as large a volume of AF as possible (at least 1 mL) in DNA extractions. This is reflected in the methodologies of Han et al. (15) and Markenson et al. (11), who extracted DNA from 1 to 2.5 mL volumes of AF, respectively. The authors of the first study were able to detect bacterial DNA via 16S rDNA PCR in 100% of culture-positive AF samples from cases of PTB. In addition, this study detected bacterial DNA in 17% of culture-negative AF samples and using molecular cloning, detected additional bacterial taxa in 9/16 culture-positive cases. The authors of the second study detected 30 PCR-positive cases of bacterial DNA in AF from 54 pregnancies with preterm labor compared to 5 cases of using only culture. In the case of large volumes of AF, bacterial cells could be pelleted in residual supernatant for subsequent DNA extraction. This would likely enhance the ability of PCR to detect organisms present in very low titers.

**Figure 1:**
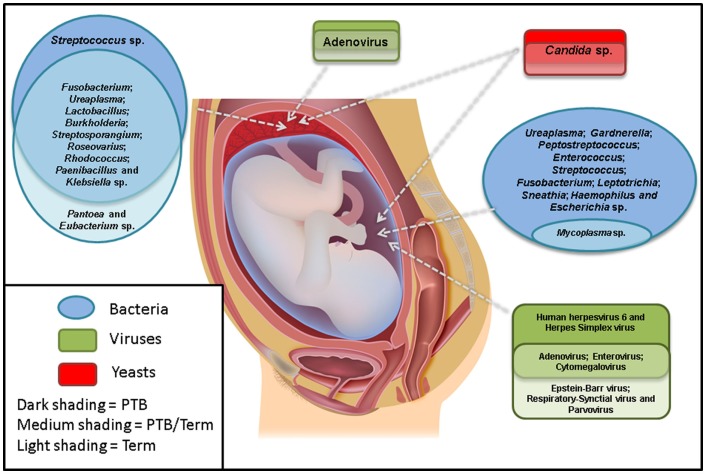
**The most commonly detected microorganisms in the amniotic fluid and placenta from preterm and term pregnancies**. Studies were only included if there were well-defined preterm and/or term cohorts.

Overall, the most commonly associated organisms with AF infection and PTB include *U. parvum*, *U. urealyticum*, *Mycoplasma hominis*, *Gardnerella vaginalis*, *Peptostreptococcus* sp., *Enterococcus* sp., *Streptococcus* sp. (particularly *S. agalactiae*), *F. nucleatum*, *Leptotrichia* sp., *S. sanguinegens*, *Haemophilus influenzae*, and *Escherichia coli* (Figure 1). However, *Ureaplasma* sp. are by far the most commonly detected organism in AF from preterm pregnancies (23) and the greatest body of evidence exists suggesting a causal association between their presence in the AF and subsequent PTB (24). Of particular significance is the case–control study by Gerber et al. (25), who described a significant association between presence of *Ureaplasma* sp. DNA in second trimester AF and subsequent preterm labor. Similar associations have been described by Yoon et al. (26, 27) and Oh et al. (28) in case-specific studies. In contrast, within AF from women who delivered at term, detection rates for *Ureaplasma* sp. ranging from 0 (20) to 5.3% (25) have been described. A common colonizer of the vagina (24), this is believed to be the major reservoir for AF infection. However to date, no studies have been able to describe why only certain women vaginally colonized by *Ureaplasma* sp. deliver preterm and others do not. Recent work in a murine model by Racicot et al. (29) has offered a new viewpoint on this topic and will be discussed later in this review.

### Viruses

During pregnancy, women are at a much greater risk of viral infection. For example, the mortality rate associated with influenza in pregnant women during the Spanish flu pandemic of 1918 was between 50 and 75% (30, 31). Unsurprisingly, numerous viral taxa have been previously described in the AF. Examples of these include rubella virus (32), varicella–zoster virus (VZV) (33), human immunodeficiency virus (34), adenovirus (35, 36), cytomegalovirus (CMV) (35, 36), herpes simplex virus (HSV) (36), human parvovirus (36), Epstein–Barr virus (EBV) (35), enterovirus (35, 37), and respiratory syncytial virus (35). All of these have been identified through either viral culture or targeted molecular assays as, unlike bacteria, at present there is no gene conserved throughout viral genera with sufficient variable regions that enables taxonomic classification. This factor dramatically limits our knowledge of the contribution of viruses to the AF microbiome.

Despite these difficulties, a small number of studies have examined the potential association between the presence of viral nucleic acids in the AF and subsequent PTB (Figure 1). The excellent review article by DiGiulio (7) summarized three such studies by Wenstrom et al. (36), Baschat et al. (35), and Miller et al. (38), all of which found a range of viral taxa in the AF of women, but did not report any association between the presence of any specific virus and negative pregnancy outcome.

More recently, Gervasi et al. (39) analyzed 729 mid-trimester AF samples for the presence of adenoviruses, HSV, VZV, human herpesvirus 6 (HHV6), CMV, EBV, parvovirus B19, and enteroviruses. They reported the presence of viral nucleic acids in 16/729 samples (2.2%) with HHV6 being the most prevalent (7 cases), followed by CMV (6 cases), parvovirus B19 (2 cases), and EBV (1 case). No cases of HSV, VZV, enteroviruses, or adenoviruses were reported. The complete absence of adenoviruses in this cohort is in stark contrast to the earlier work of Wenstrom et al. (36) and Baschat et al. (35), who both reported adenoviruses as the most common amongst viral nucleic acid-positive AF samples (9/14 and 37/44 positive AF samples, respectively). Regardless, similar to previous studies, Gervasi et al. (39) also reported no significant association between the presence of any specific viral nucleic acid in mid-trimester AF and negative pregnancy outcome.

Romero et al. (22) utilized a novel molecular method that combined PCR with electrospray ionization time-of-flight mass spectrometry to examine AF from 142 women in preterm labor with intact membranes for the viruses, HSV-1 and -2, VZV, EBV, CMV, Kaposi’s sarcoma-associated herpes virus, human adenoviruses, human enteroviruses, BK polyomavirus, JC polyomavirus, and parvovirus B19. Viral DNA was only identified in two cases, both of which were identified as enteroviruses.

Two additional recent studies examined any relationship between the presence of viral nucleic acid in AF and the clinical phenotype of preterm premature rupture of membranes (PPROM). The first of these was unable to detect any nucleic acid from HSV-1, HSV-2, adenovirus, adeno-associated virus-2, CMV, parvovirus B19, human papilloma viruses, and enteroviruses in the AF of 13 women with PPROM (40). Similarly, Bopegamage et al. (41) were only able to detect a single viral nucleic acid-positive AF sample from a cohort of 174 women with PPROM. In this case, the positive sample contained CMV DNA, but the study also tested for HSV, parvovirus B19, adenovirus, enterovirus, and human parechovirus.

Although these studies appear to paint a clear picture that no association exists between the presence of viruses and subsequent PTB, there are two important considerations to make. First, as mentioned above, our knowledge of viral taxa in AF is greatly limited by the lack of suitable viral phylogenetic markers similar to the bacterial 16S rRNA gene. At present, the only technology available to examine the viral microbiome that does not involve the use of targeted molecular assays is whole genome shotgun metagenomics. This is a rapidly expanding area in viral detection and identification and has resulted in the discovery of novel viral pathogens (42, 43). There is high potential for the application of this technology to examine the role of viral infection in cases of PTB. Second, if one is to adopt the view that PTB is actually a polymicrobial condition, as has been suggested in recent literature, then the view of whether viruses play a role can easily be revisited. Recent research by Racicot et al. (29) has added an interesting twist to this topic and will be discussed later regarding PTB as a polymicrobial disease.

### Fungi and yeasts

Similar to viruses, our knowledge of fungi and yeasts in the uterine cavity at present is largely limited to culture and targeted molecular assays. Unlike viruses, however, this is not through lack of suitable phylogenetically informative genes. In fact, in fungi and yeasts several such targets exist, the 18S, 5.8S, and 28S rRNA genes as well as the internal transcribed spacer regions (ITS1 and ITS2) (44). The major limiting factor of these targets compared to the bacterial 16S rRNA gene, however, is that the reference databases containing sequence identifications are significantly less populated than those for bacteria. In addition, the 18S rRNA gene is also present in the human genome, which presents a problem with NGS technologies in terms of the generation of unwanted amplicons utilizing sequencing reagents. This is likely to be of much more significance in samples with very small fungal/yeast content, where the levels of human DNA in an extract may greatly outnumber fungal/yeast DNA.

The only study to date that we are aware of which has employed a broad-range PCR approach to elucidating the fungal microbiome in AF is that of DiGiulio et al. (14). This study utilized the 18S–28S rRNA genes and only detected a single positive sample from 166 patients, which was identified as *Candida albicans*.

Although they are one of the most common organisms found in the vagina of pregnant women (45, 46) only a small number of studies have investigated *Candida* sp. as a potential source of AF infection and PTB. These have included both culture (47, 48) and molecular-based (14, 18) studies of women in preterm labor with intact membranes and women with PPROM, with prevalence rates varying between 0 and 1.2% for the first clinical phenotype and 0–5% for the second clinical phenotype. Numerous studies have also reported a distinct association between the presence of an intra-uterine device (IUD) during pregnancy and *Candida* sp. intra-uterine infection (7), with one study describing this phenomenon in 31.1% of pregnancies where an IUD was present vs. 6.3% where there was no IUD (49).

Unlike organisms more commonly associated with intra-uterine infection, the consequences of *Candida* sp. in the uterine cavity are typically severe. For example, Payne et al. (50) used a pregnant sheep model to demonstrate that colonization of the amniotic cavity by *C. albicans* causes severe uterine inflammation and subsequent fetal injury. Once acquired by a preterm neonate, *Candida* sp. colonization can rapidly progress to invasive candidiasis, a condition that is frequently associated with low birthweight, prematurely born infants. It has a high mortality and morbidity rate and is reported to be the second most common fatal infection associated with preterm infants (51).

At present the fungal/yeast microbiome of the uterine cavity is largely underestimated and our knowledge appears to be limited to *Candida* sp. (Figure 1). Microbiome investigations of other human body sites utilizing the aforementioned phylogenetically informative targets have revealed previously undescribed diversity, although levels are typically several magnitudes lower than that for bacterial studies (44). For example, in a study of the oral fungal/yeast microbiome, Ghannoum et al. (52) identified *Candida* species as the most common (present in 75% of participants), followed by *Cladosporium* sp. (65%), *Aureobasidium* sp., *Saccharomycetales* sp. (50% for both), *Aspergillus* sp. (35%), *Fusarium* sp. (30%), and *Cryptococcus* sp. (20%). Of more relevance to PTB, LaTuga et al. (53) detected fungal/yeast DNA in 7/11 stool specimens from extremely low birthweight infants. Genera identified included *Candida* sp., *Cladosporium* sp., *Clavisporas* sp., *Cryptococcus* sp., and *Saccharomyces* sp.

Our knowledge of fungi/yeasts that invade the uterine cavity is likely to substantially improve in future years through utilization of NGS technologies and conserved fungal/yeast genes. Comparison of such microbial communities with pregnancy outcome will allow an informed decision of the role that fungi/yeasts play in PTB.

## Placental Microbiome

Bacterial colonization of the placenta has been reported previously on several occasions (Table 1). Although numerous studies have suggested colonization as a result of prior fetal membrane and AF infection, an increasing number of studies are reporting the presence of bacteria representative of the oral microbiota in the placenta and suggesting hematogenous transfer as the route of colonization. This will be discussed in detail later in this review.

### Bacteria

One of the best studies to truly document bacterial colonization of the placenta is that by Stout et al. (54), who hypothesized that the maternal basal plate of the placenta may be a reservoir for bacteria associated with negative pregnancy outcomes. In a study of 195 women, they reported Gram positive and negative intracellular bacteria of a range of morphologies (filamentous, cocci, rods, and spirochetes) in the basal plates from 27% of all placentas. In the case of preterm vs. term deliveries, there was a significant association identified between presence of bacteria in the basal plate and delivery at <28 weeks gestational age (GA) (54.5 vs. 26.7%), however, at preterm GAs >28 weeks GA, this significance was lost. Interestingly, the study also reported the presence of bacteria in placentas in the absence of clinical or pathologic chorioamnionitis, potentially indicating placental bacterial colonization through hematogenous mechanisms. Unfortunately, the authors did not go beyond Gram and cell morphology classification in this study, which would have been greatly enhanced with the use of laser capture micro-dissection to isolate specific cell morphologies and identify these using 16S rDNA techniques.

The most recent placental microbiome study applied cutting edge NGS methodologies to elucidate the bacterial microbiota of the placenta. Aagaard et al. (3) described a unique microbiome that bore similarity to oral taxa from non-pregnant subjects, specifically to the bacterial microbiota of the tongue, tonsils, and gingival plaques as previously described by the Human Microbiome Project consortium (55, 56). Using whole genome shotgun metagenomics, the most frequently detected sequences belonged to *E. coli* and the genus *Escherichia* sp; *Prevotella tannerae*, *Bacteroides* sp., *Streptomyces avermitilis*, *Propionibacterium acnes*, *Rhodococcus erythropolis*, *Neisseria polysaccharea*, *Neisseria lactamica*, and *Fusobacterium* sp. sequences were also detected in lower numbers. Of substantial interest amongst these sequence identifications is that *E. coli* appears to dominate placental bacterial communities. Aagaard et al. (3) suggested that the source of colonization may be infant meconium, a highly plausible theory considering recent studies showing the high abundance of *E. coli* in meconium from neonates (57, 58). Another potential source may be the maternal gut, where *E. coli* is a common resident. This would entirely depend on the ability of *E. coli* to cross the mucosal barrier of the intestine. A prime example of this is *Listeria monocytogenes*, which following passage across the intestine is able to spread hematogenously to various body sites, particularly the fetoplacental unit (57).

Aagaard et al. (3) also used a 16S rDNA approach to characterize the bacterial microbiota of a larger number of placental samples and looked for associations between these and PTB. Interestingly, they detected an enrichment of sequences associated with *Burkholderia* sp. in samples from women who delivered preterm. This genus contains known respiratory pathogens such as the *Burkholderia cepacia* complex. Other organisms detected included *Streptosporangium* sp., *Roseovarius* sp., *Rhodococcus* sp., *Paenibacillus* sp., *Klebsiella* sp., and *Anaeromyxobacter* sp. (Figure 1). These taxa are quite different to those described by Onderdonk et al. (59), who reported positive cultures for 696/1365 placentas from pregnancies 23–27 weeks GA. The most commonly reported organisms were *Actinomyces* sp., *Streptococcus* sp., *Corynebacterium* sp., *E. coli*, *Lactobacillus* sp., *M. hominis*, *Peptostreptococcus* sp., *Prevotella bivia*, *Propionibacterium* sp., coagulase-negative Staphylococci, *Bacteroides* sp., *G. vaginalis*, and *Ureaplasma* sp.

Although the previously discussed study by Aagaard et al. (3), to our knowledge, represents the only “true” microbiome analysis of the placenta at present, numerous studies have documented a range of other bacteria in this organ. For example, a number of intracellular bacteria are known to colonize the placenta and are associated with negative pregnancy outcomes. These include *L. monocytogenes* (60), *Coxiella burnetii* (60, 61), *Chlamydia trachomatis* (60, 62), *Waddlia chondrophila* (60, 63), and *Parachlamydia acanthamoebae* (60).

A more recent study by Queiros da Mota et al. (64) reported 73 cases of positive bacterial culture from 376 placentas. Of these cases, 48 were described as monomorphic and half of the placentas with positive cultures were from preterm deliveries. They described the presence of a range of bacteria, dominated by Gram positive cocci and bacilli and Gram negative bacilli. A number of anaerobes of these same morphology were also present, particularly Gram negative bacilli. The most interesting aspect of this study, however, was the correlation between histological chorioamnionitis and placental bacterial culture. Of the 73 culture-positive cases, 28 occurred in the presence of chorioamnionitis, while 45 did not. This adds some support to the theory that not all cases of bacterial colonization are indeed infection and as suggested by Aagaard et al. (3) that the placenta may indeed harbor its own unique microbiome.

Our knowledge of the bacterial placental microbiome is likely to substantially improve in coming years with the increased application of NGS-based technologies. Data generated by such studies combined with detailed patient histories is likely to significantly enhance our knowledge of the role the placenta plays as a source of bacterial colonization and how this colonization impacts on pregnancy outcome.

### Viruses

Only a small number of studies have attempted to document the presence of viruses in the placenta. The first of these was a study by Srinivas et al. (65) that looked at singleton pregnancies presenting with a spontaneous second trimester pregnancy loss secondary to PPROM, premature labor, or cervical insufficiency. The authors detected significantly more viral nucleic acid in cases (79%) compared to controls (second trimester induction of labor for congenital anomalies or maternal medical indications) (44%). The major viruses detected were CMV and HPV.

Several years later, Tsekoura et al. (66) examined 71 preterm and 122 full term placentas for the presence of adenovirus DNA and reported its presence in 40.8 and 20.5% of preterm and term cases, respectively (Figure 1). This was a significant finding. In addition, they also documented a significant increase in cases of histological chorioamnionitis in preterm adenovirus-positive placentas when compared to both preterm adenovirus-negative placentas and term adenovirus-positive placentas (75 vs. 36 vs. 19%, respectively).

Perhaps, the most important study looking at viruses in the placenta is that by Cardenas et al. (30), which outlined the importance of viral placental infection in a murine model. This study used murine herpesvirus-68 (MHV-68)-infected pregnant mice to show that viral infection of the placenta can elicit a fetal inflammatory response and that such an infection also may sensitize the mother to bacterial endotoxin and in turn, preterm labor. The authors injected LPS into MHV-68-infected mice in a dose that was known to have a modest effect on pregnancy outcome (20 μg/kg). All MHV-68/LPS animals subsequently delivered in <24 h post-LPS injection compared with only 29% of LPS-only animals. In addition, there was vaginal bleeding and a 100% fetal death rate observed in all MHV-68/LPS cases compared to none in LPS-only animals.

### Fungi and yeasts

As with the AF, with the exception of *Candida* sp., there is a complete dearth of information regarding the fungal/yeast microbiome of the placenta (Figure 1). There have been several case reports documenting placental *Candida* sp. infections, in particular those arising from cutaneous congenital candidiasis (67–70). This is an extremely rare disease (<100 published cases) that typically occurs secondary to *Candida* sp. chorioamnionitis. The phenotype is characterized by the presence of white microabscesses on the placenta and umbilical cord and a generalized rash on the infant shortly after birth (69).

## Pathways to Microbial Colonization of the Uterine Cavity

The excellent review article by Goldenberg et al. (1) on the epidemiology and causes of PTB proposed four major routes of how microbial organisms are able to invade the uterine cavity. These were vertical ascension from the vagina; retrograde through the abdominal cavity, introduction through invasive procedures such as amniocentesis and hematogenously from the placenta. It has been well established that the major source of intra-uterine colonization is vertical ascension from the vagina (1), and this is largely believed to occur during the second trimester, although the actual timing is unknown and it is likely that this will vary between individual pregnancies.

The evidence currently supporting hematogenous spread of microbes however, is a contentious area that needs to be viewed carefully. There are increasing reports that bacteria, specifically those from the oral cavity, are able to spread hematogenously from the maternal bloodstream to the uterine cavity (5). This is further supported by apparent associations between periodontal disease and PTB (71, 72), although this association is also contentious with numerous studies (73), including a large randomized-controlled trial (74) finding that treatment of periodontal disease during pregnancy does not reduce the rate of PTB.

The best evidence supporting hematogenous spread of oral bacteria to the uterine cavity is provided through numerous studies by Han et al. The first of these was in a murine model where mice received an intravenous (IV) injection of live *F. nucleatum*. This subsequently spread to the uterus and resulted in negative pregnancy outcomes (75). Following this, Han et al. (4) attempted to show transfer of an uncultured *Bergeyella* sp. strain from the oral cavity to the AF in a human case of PTB. The study identified the organism based upon its 16S-23S rDNA sequence and concluded that as the sequence homology was identical between the AF and sub gingival plaque sites that this demonstrated oral to AF transfer. Han et al. (76) then reported a case study of a woman with pregnancy-associated gingivitis who experienced an upper respiratory tract infection and subsequent stillbirth. *F. nucleatum* was isolated from both the placenta and infant and subsequent 16S–23S rDNA analysis of vaginal and rectal swabs failed to detect the presence of the organism. However, it was detected in the sub and supragingival plaques, and in the case of the subgingival plaque, the apparent identical clone was detected based upon sequence similarity. Unfortunately, the case study did not note the timeframe associated with still birth to collection of vaginal/rectal samples, which is important for validating the failure to detect *F. nucleatum* in these. These case studies offer the most robust information to date on potential oral-uterine bacterial transfer in humans. Further work by Fardini et al. (5) has shown potential transfer of a range of oral bacterial species to the murine placenta through IV inoculation; however, the method of detection in the placenta was DNA-based as opposed to culture. The reason that DNA-based studies such as these are contentious is that although they do indeed show the presence of microbial DNA in the uterine cavity that corresponds with that of species synonymous with the oral microbiota, they do not show the presence of viable microbial cells. Recently, it has been well publicized that cell-free fetal DNA is trafficked out of the placenta and into the maternal circulation, where it is readily detectable during pregnancy (77–81). Based upon this, it would also be plausible that lower molecular weight, microbial DNA can cross from the maternal bloodstream to the uterine cavity and vice-versa. Detection of microbial DNA in these samples at best demonstrates that such DNA can be spread from the maternal bloodstream to the uterine cavity. This said, the presence of microbial DNA in the uterine cavity alone may be enough to activate inflammatory responses that culminate in preterm labor.

Although work to date offers increasingly promising evidence that the oral microbiota can infect the uterine cavity through hematogenous transfer, further work is required to definitively uncover their role in intra-uterine infection. Due to inherent difficulties with culture of fastidious organisms present at these sites, it is increasingly important that molecular detection/characterization protocols are employed that represent the viable microbiota in these samples as opposed to lysed cells or free-circulating DNA. Such methodologies are discussed below.

## Microbial Cell Viability, the Microbiome, and Clinical Translation

Although current research to elucidate the various microbiomes of the uterine environment have been limited to DNA-based approaches, the issue of how relevant DNA detection is on a clinical level has been present for many years. It has long been known that DNA is a stable molecule and can persist for weeks following microbial cell death (82). Wang and Levin suggested that the inability of DNA-based PCR assays to differentiate between non-viable and viable cells was a major limitation of this technology (83). Applying this to microbiome-level studies, which may be characterizing dynamic systems over several time points, detection of viable cells is critical to documenting microbial succession. In addition, in confined environments such as the uterus, where there is poor clearance of cellular material and particularly, in these scenarios following antibiotic usage; non-viable organisms and extracellular DNA can contribute significantly to molecular analyses (84).

Some studies have attempted to remedy this by utilizing the amplification of RNA instead of DNA, which degrades rapidly after cell death and in particular, using messenger RNA targets as this is a highly unstable molecule and is only produced by metabolically active cells (85–88). The major disadvantage to this approach, however, lies in the inherent difficulties associated with isolating RNA from samples, including the need for stringent sample storage conditions following sample collection, in addition to sample processing regimes to prevent RNA degradation (86). For example, RNA-degrading enzymes, ribonucleases (RNases), can rapidly degrade RNA if not promptly inhibited. The human skin is a prime example of how RNases can be accidentally introduced into samples (89).

In terms of using RNA for microbiome characterization, this instability is the major limitation, as even minor degradation of nucleic acid can potentially result in loss of characterization of the total viable microbial community in a given sample, especially that of organisms present in low cell titers.

A potential solution that addresses the issue of cell viability in DNA-based methodologies and may be highly applicable to microbiome-level studies is that of viability PCR (vPCR). This technology utilizes membrane-impermeable dyes, either ethidium monoazide (EMA) (90) or the more recent and preferred propidium monoazide (PMA) (91). Samples are pre-treated with the chosen dye, which is unable to cross an intact cell wall. In cases, where the integrity of the microbial cell wall has been lost, the dye is able to intercalate into the cell’s DNA, which results in covalent cross-linkage after exposure to strong visible light. Cross-linked DNA is subsequently blocked from PCR amplification in downstream analyses (86). This technology has been applied to bacteria (92, 93), fungi (94), viruses (95), yeasts (96), and protozoa (97) on many previous occasions and has also been used in both environmental (98) and clinical (84) microbiome analyses. A detailed review of this technology is provided by Fittipaldi et al. (86).

However, although vPCR certainly has the potential to yield clinically relevant microbiome data, careful validation is first needed for some of the key microorganisms associated with PTB. In particular, organisms of the Class Mollicutes, including all *Ureaplasma* and *Mycoplasma* sp. do not possess a true bacterial cell wall. Their nucleic acid is instead protected by a triple layered membrane and its permeability to EMA or PMA is currently unknown. An additional consideration that is highly relevant to microbiome studies is that surrounding the buffer used to resuspend swab-collected samples. It is very important that the buffer itself does not result in cell lysis. For example, an alkaline pH may result in significant loss of viability of *Lactobacillus* sp. cells from a vaginal swab.

## Preterm Birth: A Polymicrobial Disease?

Many studies, particularly since the increase in NGS-based microbiome work, have emphasized the importance of assessing PTB as a polymicrobial disease, a large number of which are summarized in Table 1, in addition to the review by DiGiulio et al. (7). However, in the context of most of these studies, the word polymicrobial is used to imply the presence of two or more bacterial species. A more appropriate term to describe such an infection would be “polybacterial. ” In a disease context, the word “polymicrobial” is best used to describe diseases involving multiple infectious agents (99). As such, a polymicrobial infection may entail the initial presence of a virus, which creates a favorable environment for a secondary bacterial or fungal infection or vice-versa.

Evidence of the importance of viewing polymicrobial disease in this way is provided by Racicot et al. (29) who recently conducted an elegant study demonstrating how a viral infection during pregnancy may compromise the antibacterial defenses of the cervix, prompting a secondary bacterial infection of the uterine cavity. These authors demonstrated that the cervix in mice shows resistance to bacterial infection with *E. coli* during pregnancy, but not in non-pregnant animals. Extending this further, they replicated the same experiment using the most commonly observed organism from preterm pregnancies, *Ureaplasma* sp., and reported the same result. Having previously shown that infecting pregnant mice with a virus, MHV-68, predisposes the animals to the effects of bacterial endotoxin, but viral infection itself does not induce preterm labor (30), they went on to test whether a systemic viral infection could alter the ability of the cervix/uterine cavity to resist bacterial infection. Following an intraperitoneal injection of MHV-68 into pregnant and non-pregnant mice, they showed that 7 days post-injection the virus was observed at similar concentrations in the spleen of both pregnant and non-pregnant animals, but was only present in the cervix of pregnant mice. They subsequently suggested that pregnancy may render the cervix susceptible to a viral infection. After administering *Ureaplasma* sp. intravaginally to MHV-68-infected and non-infected pregnant mice, they reported significantly higher amounts of *Ureaplasma* sp. nucleic acid in the decidua and lymphoid aggregates of MHV-68-infected mice compared to non-infected. The authors suggested that a viral infection during pregnancy can alter the ability of the female reproductive tract to defend against an ascending bacterial infection (29).

Although this work was carried out in mice and potentially may not apply to humans due to physiological differences in the cervix and pregnancy in general, it still provides substantial evidence that future microbiome studies of the uterine cavity need to not only focus on bacteria, but also other organisms including viruses and fungi/yeasts, and document any relationship between these and negative pregnancy outcomes.

## Conclusion

Our knowledge of the microbiome of the uterine cavity has been greatly enhanced since the widespread use of molecular microbiological techniques, particularly 16S rDNA phylogenetics, which have uncovered numerous bacterial taxa not previously described. Bacteria, particularly *Ureaplasma* sp. and *Fusobacterium* sp. appear to be most significantly associated with negative pregnancy outcomes when present in the uterine compartment. Although viruses are also present and on their own do not appear to be significant, when combined with a bacterial infection they may contribute significantly to PTB. Viral infection of the placenta, however, does appear to be associated with negative pregnancy outcomes. Our knowledge of fungi/yeasts that colonize the uterine cavity is currently limited to yeasts, specifically *Candid*a sp. Further research effort is required to characterize the fungal microbiome of the uterine cavity using conserved fungal/yeast genes. These data combined with existing data on bacteria and viruses are likely to shed further light on the polymicrobial nature of intra-uterine infections.

Although current microbiome-based studies have contributed valuable data to our knowledge of intra-uterine infection, the application of these data to clinical scenarios is currently limited due to cell viability issues surrounding DNA-based analyses. Future microbiome-based studies, especially those attempting to document hematogenous spread of viable microbial cells from various body sites to the uterine cavity, should adopt molecular approaches that either:

(1) Utilize RNA-based characterization of a given microbial community using known conserved genes (for instance, the 16S rRNA gene in bacteria), coupled with strict sample collection and processing regimes so as to inhibit the activity of RNases.
(2) Maintain current DNA-based characterization approaches, but implement vPCR procedures to inhibit amplification of DNA from non-viable cells.

These approaches are likely to be of particular relevance if/when microbiome-based NGS approaches are introduced into clinical diagnostic laboratories.

## Conflict of Interest Statement

The authors declare that the research was conducted in the absence of any commercial or financial relationships that could be construed as a potential conflict of interest.

## References

[1] Goldenberg RL Culhane JF Iams JD Romero R Epidemiology and causes of preterm birth. Lancet (2008) 371(9606):75–84 10.1016/S0140-6736(08)60074-4 18177778 PMC7134569

[2] Goldenberg RL Hauth JC Andrews WW Intrauterine infection and preterm delivery. N Engl J Med (2000) 342(20):1500–7 10.1056/NEJM200005183422007 10816189

[3] Aagaard K Ma J Antony KM Ganu R Petrosino J Versalovic J. The placenta harbors a unique microbiome. Sci Transl Med (2014) 6(237):237ra65. 10.1126/scitranslmed.3008599 24848255 PMC4929217

[4] Han YW Ikegami A Bissada NF Herbst M Redline RW Ashmead GG. Transmission of an uncultivated *Bergeyella* strain from the oral cavity to amniotic fluid in a case of preterm birth. J Clin Microbiol (2006) 44(4):1475–83. 10.1128/JCM.44.4.1475-1483.2006 16597879 PMC1448680

[5] Fardini Y Chung P Dumm R Joshi N Han YW. Transmission of diverse oral bacteria to murine placenta: evidence for the oral microbiome as a potential source of intrauterine infection?†. Infect Immun (2010) 78(4):1789–96. 10.1128/IAI.01395-09 20123706 PMC2849412

[6] Harris JW Brown H Bacterial content of the uterus at cesarean section. Am J Obstet Gynecol (1927) 13:133.

[7] DiGiulio DB. Diversity of microbes in amniotic fluid. Semin Fetal Neonatal Med (2012) 17(1):2–11. 10.1016/j.siny.2011.10.001 22137615

[8] Mendz GL Kaakoush NO Quinlivan JA. Bacterial aetiological agents of intra-amniotic infections and preterm birth in pregnant women. Front Cell Infect Microbiol (2013) 3:58. 10.3389/fcimb.2013.00058 24137568 PMC3797391

[9] Combs CA Gravett M Garite TJ Hickok DE Lapidus J Porreco R Amniotic fluid infection, inflammation, and colonization in preterm labor with intact membranes. Am J Obstet Gynecol (2014) 210(2):e1–15. 10.1016/j.ajog.2013.11.032 24274987

[10] Jalava J Mantymaa ML Ekblad U Toivanen P Skurnik M Lassila O Bacterial 16S rDNA polymerase chain reaction in the detection of intra-amniotic infection. Br J Obstet Gynaecol (1996) 103(7):664–9. 10.1111/j.1471-0528.1996.tb09835.x 8688393

[11] Markenson GR Martin RK Tillotson-Criss M Foley KS Stewart RS Jr Yancey M. The use of the polymerase chain reaction to detect bacteria in amniotic fluid in pregnancies complicated by preterm labor. Am J Obstet Gynecol (1997) 177(6):1471–7. 10.1016/S0002-9378(97)70093-0 9423753

[12] Hitti J Riley DE Krohn MA Hillier SL Agnew KJ Krieger JN Broad-spectrum bacterial rDNA polymerase chain reaction assay for detecting amniotic fluid infection among women in premature labor. Clin Infect Dis (1997) 24(6):1228–32. 10.1086/513669 9195088

[13] Gardella C Riley DE Hitti J Agnew K Krieger JN Eschenbach D. Identification and sequencing of bacterial rDNAs in culture-negative amniotic fluid from women in premature labor. Am J Perinatol (2004) 21(6):319–23. 10.1055/s-2004-831884 15311367

[14] DiGiulio DB Romero R Amogan HP Kusanovic JP Bik EM Gotsch F Microbial prevalence, diversity and abundance in amniotic fluid during preterm labor: a molecular and culture-based investigation. PLoS One (2008) 3(8):e3056. 10.1371/journal.pone.0003056 18725970 PMC2516597

[15] Han YW Shen T Chung P Buhimschi IA Buhimschi CS. Uncultivated bacteria as etiologic agents of intra-amniotic inflammation leading to preterm birth. J Clin Microbiol (2009) 47(1):38–47. 10.1128/JCM.01206-08 18971361 PMC2620857

[16] Jones HE Harris KA Azizia M Bank L Carpenter B Hartley JC Differing prevalence and diversity of bacterial species in fetal membranes from very preterm and term labor. PLoS One (2009) 4(12):e8205. 10.1371/journal.pone.0008205 19997613 PMC2785424

[17] DiGiulio DB Gervasi MT Romero R Vaisbuch E Mazaki-Tovi S Kusanovic JP Microbial invasion of the amniotic cavity in pregnancies with small-for-gestational-age fetuses. J Perinat Med (2010) 38(5):495–502. 10.1515/JPM.2010.076 20482466 PMC2962935

[18] DiGiulio DB Gervasi M Romero R Mazaki-Tovi S Vaisbuch E Kusanovic JP Microbial invasion of the amniotic cavity in preeclampsia as assessed by cultivation and sequence-based methods. J Perinat Med (2010) 38(5):503–13. 10.1515/JPM.2010.078 20482470 PMC3325506

[19] DiGiulio DB Romero R Kusanovic JP Gomez R Kim CJ Seok K Prevalence and diversity of microbes in the amniotic fluid, the fetal inflammatory response, and pregnancy outcome in women with preterm prelabor rupture of membranes. Am J Reprod Immunol (2010) 64(1):38–57. 10.1111/j.1600-0897.2010.00830.x 20331587 PMC2907911

[20] Marconi C de Andrade Ramos BR Peracoli JC Donders GG da Silva MG. Amniotic fluid interleukin-1 beta and interleukin-6, but not interleukin-8 correlate with microbial invasion of the amniotic cavity in preterm labor. Am J Reprod Immunol (2011) 65(6):549–56. 10.1111/j.1600-0897.2010.00940.x 21214658

[21] Wang X Buhimschi CS Temoin S Bhandari V Han YW Buhimschi IA. Comparative microbial analysis of paired amniotic fluid and cord blood from pregnancies complicated by preterm birth and early-onset neonatal sepsis. PLoS One (2013) 8(2):e56131. 10.1371/journal.pone.0056131 23437088 PMC3577789

[22] Romero R Miranda J Chaiworapongsa T Chaemsaithong P Gotsch F Dong Z A novel molecular microbiologic technique for the rapid diagnosis of microbial invasion of the amniotic cavity and intra-amniotic infection in preterm labor with intact membranes. Am J Reprod Immunol (2014) 71(4):330–58. 10.1111/aji.12189 24417618 PMC3954440

[23] Waites KB Schelonka RL Xiao L Grigsby PL Novy MJ. Congenital and opportunistic infections: *Ureaplasma* species and *Mycoplasma hominis*. Semin Fetal Neonatal Med (2009) 14(4):190–9. 10.1016/j.siny.2008.11.009 19109084

[24] Capoccia R Greub G Baud D. *Ureaplasma urealyticum*, *Mycoplasma hominis* and adverse pregnancy outcomes. Curr Opin Infect Dis (2013) 26(3):231–40. 10.1097/QCO.0b013e328360db58 23587772

[25] Gerber S Vial Y Hohlfeld P Witkin SS. Detection of *Ureaplasma urealyticum* in second-trimester amniotic fluid by polymerase chain reaction correlates with subsequent preterm labor and delivery. J Infect Dis (2003) 187(3):518–21. 10.1086/368205 12552439

[26] Yoon BH Romero R Kim M Kim EC Kim T Park JS Clinical implications of detection of *Ureaplasma urealyticum* in the amniotic cavity with the polymerase chain reaction. Am J Obstet Gynecol (2000) 183(5):1130–7. 10.1067/mob.2000.109036 11084554

[27] Yoon BH Chang JW Romero R. Isolation of *Ureaplasma urealyticum* from the amniotic cavity and adverse outcome in preterm labor. Obstet Gynecol (1998) 92(1):77–82. 10.1016/S0029-7844(98)00122-7 9649098

[28] Oh KJ Lee SE Jung H Kim G Romero R Yoon BH. Detection of ureaplasmas by the polymerase chain reaction in the amniotic fluid of patients with cervical insufficiency. J Perinat Med (2010) 38(3):261–8. 10.1515/JPM.2010.040 20192887 PMC3085903

[29] Racicot K Cardenas I Wunsche V Aldo P Guller S Means RE Viral infection of the pregnant cervix predisposes to ascending bacterial infection. J Immunol (2013) 191(2):934–41. 10.4049/jimmunol.1300661 23752614 PMC4153356

[30] Cardenas I Means RE Aldo P Koga K Lang SM Booth CJ Viral infection of the placenta leads to fetal inflammation and sensitization to bacterial products predisposing to preterm labor. J Immunol (2010) 185(2):1248–57. 10.4049/jimmunol.1000289 20554966 PMC3041595

[31] Nuzum JW Pilot I Stangl FH Bonar BE 1918 pandemic influenza and pneumonia in a large civil hospital. IMJ Ill Med J (1976) 150(6):612–6. 12100

[32] Van Le S Le DH Hoang HT Hoang H Nguyen NT Chu HH. Characterization of rubella virus genotypes among pregnant women in northern Vietnam, 2011-2013. J Med Virol (2014). 10.1002/jmv.24049 25111367

[33] Weisz B Book M Lipitz S Katorza E Achiron R Grossman Z Fetal outcome and amniocentesis results in pregnancies complicated by varicella infection. J Obstet Gynaecol Can (2011) 33(7):720–4. 21749748 10.1016/S1701-2163(16)34957-X

[34] Maiques V Garcia-Tejedor A Perales A Cordoba J Esteban RJ. HIV detection in amniotic fluid samples. Amniocentesis can be performed in HIV pregnant women? Eur J Obstet Gynecol Reprod Biol (2003) 108(2):137–41. 10.1016/S0301-2115(02)00405-0 12781400

[35] Baschat AA Towbin J Bowles NE Harman CR Weiner CP. Prevalence of viral DNA in amniotic fluid of low-risk pregnancies in the second trimester. J Matern Fetal Neonatal Med (2003) 13(6):381–4. 10.1080/jmf.13.6.381.384 12962262

[36] Wenstrom KD Andrews WW Bowles NE Towbin JA Hauth JC Goldenberg RL. Intrauterine viral infection at the time of second trimester genetic amniocentesis. Obstet Gynecol (1998) 92(3):420–4. 10.1016/S0029-7844(98)00210-5 9721782

[37] Reddy UM Baschat AA Zlatnik MG Towbin JA Harman CR Weiner CP. Detection of viral deoxyribonucleic acid in amniotic fluid: association with fetal malformation and pregnancy abnormalities. Fetal Diagn Ther (2005) 20(3):203–7. 10.1159/000083906 15824499

[38] Miller JL Harman C Weiner C Baschat AA. Perinatal outcomes after second trimester detection of amniotic fluid viral genome in asymptomatic patients. J Perinat Med (2009) 37(2):140–3. 10.1515/JPM.2009.027 18956964

[39] Gervasi MT Romero R Bracalente G Chaiworapongsa T Erez O Dong Z Viral invasion of the amniotic cavity (VIAC) in the midtrimester of pregnancy. J Matern Fetal Neonatal Med (2012) 25(10):2002–13. 10.3109/14767058.2012.683899 22524157 PMC3498469

[40] Naresh A Simhan H. Absence of viruses in amniotic fluid of women with PPROM: a case series. J Reprod Immunol (2012) 96(1–2):79–83. 10.1016/j.jri.2012.08.003 23021256

[41] Bopegamage S Kacerovsky M Tambor V Musilova I Sarmirova S Snelders E Preterm prelabor rupture of membranes (PPROM) is not associated with presence of viral genomes in the amniotic fluid. J Clin Virol (2013) 58(3):559–63. 10.1016/j.jcv.2013.09.013 24113293

[42] Bibby K. Metagenomic identification of viral pathogens. Trends Biotechnol (2013) 31(5):275–9. 10.1016/j.tibtech.2013.01.016 23415279

[43] Finkbeiner SR Allred AF Tarr PI Klein EJ Kirkwood CD Wang D. Metagenomic analysis of human diarrhea: viral detection and discovery. PLoS Pathog (2008) 4(2):e1000011. 10.1371/journal.ppat.1000011 18398449 PMC2290972

[44] Huffnagle GB Noverr MC. The emerging world of the fungal microbiome. Trends Microbiol (2013) 21(7):334–41. 10.1016/j.tim.2013.04.002 23685069 PMC3708484

[45] Parveen N Munir AA Din I Majeed R. Frequency of vaginal candidiasis in pregnant women attending routine antenatal clinic. J Coll Physicians Surg Pak (2008) 18(3):154–7.03.2008/JCPSP.154157 18460243

[46] Sobel JD. Vulvovaginal candidosis. Lancet (2007) 369(9577):1961–71. 10.1016/S0140-6736(07)60917-9 17560449

[47] Chaim W Mazor M Wiznitzer A. The prevalence and clinical significance of intraamniotic infection with *Candida* species in women with preterm labor. Arch Gynecol Obstet (1992) 251(1):9–15. 10.1007/BF02718273 1550392

[48] Nowakowska D Wilczynski J Szaflik K Kurnatowska A. Search for the pathogenic fungi in amniotic fluid. Wiad Parazytol (2001) 47(Suppl 1):143–6. 16897966

[49] Kim SK Romero R Kusanovic JP Erez O Vaisbuch E Mazaki-Tovi S The prognosis of pregnancy conceived despite the presence of an intrauterine device (IUD). J Perinat Med (2010) 38(1):45–53 10.1515/jpm.2009.133 19650756 PMC3418877

[50] Payne MS Kemp MW Kallapur SG Kannan PS Saito M Miura Y Intrauterine *Candida albicans* infection elicits severe inflammation in fetal sheep. Pediatr Res (2014) 75(6):716–22. 10.1038/pr.2014.35 24632681 PMC4530618

[51] Benjamin DK Jr Stoll BJ Gantz MG Walsh MC Sanchez PJ Das A Neonatal candidiasis: epidemiology, risk factors, and clinical judgment. Pediatrics (2010) 126(4):e865–73. 10.1542/peds.2009-3412 20876174 PMC3045840

[52] Ghannoum MA Jurevic RJ Mukherjee PK Cui F Sikaroodi M Naqvi A Characterization of the oral fungal microbiome (mycobiome) in healthy individuals. PLoS Pathog (2010) 6(1):e1000713. 10.1371/journal.ppat.1000713 20072605 PMC2795202

[53] LaTuga MS Ellis JC Cotton CM Goldberg RN Wynn JL Jackson RB Beyond bacteria: a study of the enteric microbial consortium in extremely low birth weight infants. PLoS One (2011) 6(12):e27858. 10.1371/journal.pone.0027858 22174751 PMC3234235

[54] Stout MJ Conlon B Landeau M Lee I Bower C Zhao Q Identification of intracellular bacteria in the basal plate of the human placenta in term and preterm gestations. Am J Obstet Gynecol (2013) 208(3):e1–7. 10.1016/j.ajog.2013.01.018 23333552 PMC3740162

[55] Human Microbiome Project Consortium. A framework for human microbiome research. Nature (2012) 486(7402):215–21. 10.1038/nature11209 22699610 PMC3377744

[56] Human Microbiome Project Consortium. Structure, function and diversity of the healthy human microbiome. Nature (2012) 486(7402):207–14. 10.1038/nature11234 22699609 PMC3564958

[57] Gosalbes MJ Llop S Valles Y Moya A Ballester F Francino MP Meconium microbiota types dominated by lactic acid or enteric bacteria are differentially associated with maternal eczema and respiratory problems in infants. Clin Exp Allergy (2013) 43(2):198–211 10.1111/cea.12063 23331561

[58] Ardissone AN de la Cruz DM Davis-Richardson AG Rechcigl KT Li N Drew JC Meconium microbiome analysis identifies bacteria correlated with premature birth. PLoS One (2014) 9(3):e90784. 10.1371/journal.pone.0090784 24614698 PMC3948723

[59] Onderdonk AB Delaney ML DuBois AM Allred EN Leviton A. Detection of bacteria in placental tissues obtained from extremely low gestational age neonates. Am J Obstet Gynecol (2008) 198(1):e1–7. 10.1016/j.ajog.2007.05.044 18166321

[60] Baud D Greub G Intracellular bacteria and adverse pregnancy outcomes. Clin Microbiol Infect (2011) 17(9):1312–22 10.1111/j.1469-0691.2011.03604.x 21884294

[61] Carcopino X Raoult D Bretelle F Boubli L Stein AQ. Fever during pregnancy: a cause of poor fetal and maternal outcome. Ann N Y Acad Sci (2009) 1166:79–89. 10.1111/j.1749-6632.2009.04519.x 19538266

[62] Baud D Goy G Jaton K Osterheld MC Blumer S Borel N Role of *Chlamydia trachomatis* in miscarriage. Emerg Infect Dis (2011) 17(9):1630–5. 10.3201/eid1709.100865 21888787 PMC3322049

[63] Baud D Goy G Osterheld MC Croxatto A Borel N Vial Y Role of Waddlia chondrophila placental infection in miscarriage. Emerg Infect Dis (2014) 20(3):460–4. 10.3201/eid2003.131019 24564950 PMC3944840

[64] Queiros da Mota V Prodhom G Yan P Hohlfheld P Greub G Rouleau C. Correlation between placental bacterial culture results and histological chorioamnionitis: a prospective study on 376 placentas. J Clin Pathol (2013) 66(3):243–8. 10.1136/jclinpath-2012-201124 23268318

[65] Srinivas SK Ma Y Sammel MD Chou D McGrath C Parry S Placental inflammation and viral infection are implicated in second trimester pregnancy loss. Am J Obstet Gynecol (2006) 195(3):797–802. 10.1016/j.ajog.2006.05.049 16949414

[66] Tsekoura EA Konstantinidou A Papadopoulou S Athanasiou S Spanakis N Kafetzis D Adenovirus genome in the placenta: association with histological chorioamnionitis and preterm birth. J Med Virol (2010) 82(8):1379–83. 10.1002/jmv.21820 20572081

[67] Delprado WJ Baird PJ Russell P Placental candidiasis: report of three cases with a review of the literature. Pathology (1982) 14(2):191–5 10.3109/00313028209061293 7099725

[68] Iwatani S Mizobuchi M Sofue T Tanaka S Sakai H Yoshimoto S Neonatal leukemoid reaction associated with *Candida albicans* chorioamnionitis. Pediatr Int (2014) 56(2):277–9. 10.1111/ped.12259 24730633

[69] Diana A Epiney M Ecoffey M Pfister RE. “White dots on the placenta and red dots on the baby”: congential cutaneous candidiasis – a rare disease of the neonate. Acta Paediatr (2004) 93(7):996–9. 10.1080/08035250410028093 15303819

[70] Ito F Okubo T Yasuo T Mori T Iwasa K Iwasaku K Premature delivery due to intrauterine *Candida* infection that caused neonatal congenital cutaneous candidiasis: a case report. J Obstet Gynaecol Res (2013) 39(1):341–3. 10.1111/j.1447-0756.2012.01938.x 22764835

[71] Shub A Wong C Jennings B Swain JR Newnham JP Maternal periodontal disease and perinatal mortality. Aust N Z J Obstet Gynaecol (2009) 49(2):130–6 10.1111/j.1479-828X.2009.00953.x 19441161

[72] Shub A Swain JR Newnham JP. Periodontal disease and adverse pregnancy outcomes. J Matern Fetal Neonatal Med (2006) 19(9):521–8. 10.1080/14767050600797749 16966119

[73] Rosa MI Pires PD Medeiros LR Edelweiss MI Martinez-Mesa J. Periodontal disease treatment and risk of preterm birth: a systematic review and meta-analysis. Cade Saude Publica (2012) 28(10):1823–33. 10.1590/S0102-311X2012001000002 23090163

[74] Newnham JP Newnham IA Ball CM Wright M Pennell CE Swain J Treatment of periodontal disease during pregnancy: a randomized controlled trial. Obstet Gynecol (2009) 114(6):1239–48. 10.1097/AOG.0b013e3181c15b40 19935025

[75] Han YW Redline RW Li M Yin L Hill GB McCormick TS. *Fusobacterium nucleatum* induces premature and term stillbirths in pregnant mice: implication of oral bacteria in preterm birth. Infect Immun (2004) 72(4):2272–9. 10.1128/IAI.72.4.2272-2279.2004 15039352 PMC375172

[76] Han YW Fardini Y Chen C Iacampo KG Peraino VA Shamonki JM Term stillbirth caused by oral *Fusobacterium nucleatum*. Obstet Gynecol (2010) 115(2 Pt 2):442–5. 10.1097/AOG.0b013e3181cb9955 20093874 PMC3004155

[77] Buysse K Beulen L Gomes I Gilissen C Keesmaat C Janssen IM Reliable noninvasive prenatal testing by massively parallel sequencing of circulating cell-free DNA from maternal plasma processed up to 24h after venipuncture. Clin Biochem (2013) 46(18):1783–6. 10.1016/j.clinbiochem.2013.07.020 23933476

[78] Nicolaides KH Wright D Poon LC Syngelaki A Gil MM. First-trimester contingent screening for trisomy 21 by biomarkers and maternal blood cell-free DNA testing. Ultrasound Obstet Gynecol (2013) 42(1):41–50. 10.1002/uog.12511 23744626

[79] Gil MM Quezada MS Bregant B Ferraro M Nicolaides KH. Implementation of maternal blood cell-free DNA testing in early screening for aneuploidies. Ultrasound Obstet Gynecol (2013) 42(1):34–40. 10.1002/uog.12504 23744609

[80] Vaiopoulos AG Athanasoula KC Papantoniou N Kolialexi A Review: advances in non-invasive prenatal diagnosis. In vivo (2013) 27(2):165–70. 23422474

[81] Simpson JL. Cell-free fetal DNA and maternal serum analytes for monitoring embryonic and fetal status. Fertil Steril (2013) 99(4):1124–34. 10.1016/j.fertnstert.2013.02.012 23499003

[82] Cenciarini-Borde C Courtois S La Scola B. Nucleic acids as viability markers for bacteria detection using molecular tools. Future Microbiol (2009) 4(1):45–64. 10.2217/17460913.4.1.45 19207099

[83] Wang S Levin RE. Discrimination of viable *Vibrio vulnificus* cells from dead cells in real-time PCR. J Microbiol Methods (2006) 64(1):1–8. 10.1016/j.mimet.2005.04.023 15932774

[84] Rogers GB Cuthbertson L Hoffman LR Wing PA Pope C Hooftman DA Reducing bias in bacterial community analysis of lower respiratory infections. ISME J (2013) 7(4):697–706. 10.1038/ismej.2012.145 23190732 PMC3603400

[85] Alifano P Bruni CB Carlomagno MS Control of mRNA processing and decay in prokaryotes. Genetica (1994) 94(2–3):157–72 10.1007/BF01443430 7534739

[86] Fittipaldi M Nocker A Codony F. Progress in understanding preferential detection of live cells using viability dyes in combination with DNA amplification. J Microbiol Methods (2012) 91(2):276–89. 10.1016/j.mimet.2012.08.007 22940102

[87] Bleve G Rizzotti L Dellaglio F Torriani S. Development of reverse transcription (RT)-PCR and real-time RT-PCR assays for rapid detection and quantification of viable yeasts and molds contaminating yogurts and pasteurized food products. Appl Environ Microbiol (2003) 69(7):4116–22. 10.1128/AEM.69.7.4116-4122.2003 12839789 PMC165170

[88] Morin NJ Gong Z Li XF. Reverse transcription-multiplex PCR assay for simultaneous detection of *Escherichia coli* O157:H7, *Vibrio cholerae* O1, and *Salmonella Typhi*. Clin Chem (2004) 50(11):2037–44. 10.1373/clinchem.2004.036814 15364889

[89] Probst J Brechtel S Scheel B Hoerr I Jung G Rammensee HG Characterization of the ribonuclease activity on the skin surface. Genet Vaccines Ther (2006) 4:4. 10.1186/1479-0556-4-4 16732888 PMC1524753

[90] Nogva HK Dromtorp SM Nissen H Rudi K. Ethidium monoazide for DNA-based differentiation of viable and dead bacteria by 5’-nuclease PCR. Biotechniques (2003) 34(4):804–8. 12703305 10.2144/03344rr02

[91] Nocker A Cheung CY Camper AK. Comparison of propidium monoazide with ethidium monoazide for differentiation of live vs. dead bacteria by selective removal of DNA from dead cells. J Microbiol Methods (2006) 67(2):310–20. 10.1016/j.mimet.2006.04.015 16753236

[92] Agusti G Codony F Fittipaldi M Adrados B Morato J. Viability determination of *Helicobacter pylori* using propidium monoazide quantitative PCR. Helicobacter (2010) 15(5):473–6. 10.1111/j.1523-5378.2010.00794.x 21083754

[93] Nam S Kwon S Kim MJ Chae JC Jae Maeng P Park JG Selective detection of viable *Helicobacter pylori* using ethidium monoazide or propidium monoazide in combination with real-time polymerase chain reaction. Microbiol Immunol (2011) 55(12):841–6. 10.1111/j.1348-0421.2011.00388.x 22004535

[94] Vesper S McKinstry C Hartmann C Neace M Yoder S Vesper A. Quantifying fungal viability in air and water samples using quantitative PCR after treatment with propidium monoazide (PMA). J Microbiol Methods (2008) 72(2):180–4. 10.1016/j.mimet.2007.11.017 18160156

[95] Fittipaldi M Rodriguez NJ Codony F Adrados B Penuela GA Morato J. Discrimination of infectious bacteriophage T4 virus by propidium monoazide real-time PCR. J Virol Methods (2010) 168(1–2):228–32. 10.1016/j.jviromet.2010.06.011 20599560

[96] Andorra I Esteve-Zarzoso B Guillamon JM Mas A. Determination of viable wine yeast using DNA binding dyes and quantitative PCR. Int J Food Microbiol (2010) 144(2):257–62. 10.1016/j.ijfoodmicro.2010.10.003 21036413

[97] Fittipaldi M Pino Rodriguez NJ Adrados B Agusti G Penuela G Morato J Discrimination of viable *Acanthamoeba castellani* trophozoites and cysts by propidium monoazide real-time polymerase chain reaction. J Eukaryot Microbiol (2011) 58(4):359–64. 10.1111/j.1550-7408.2011.00557.x 21699621

[98] Nocker A Richter-Heitmann T Montijn R Schuren F Kort R. Discrimination between live and dead cellsin bacterial communities from environmental water samples analyzed by 454 pyrosequencing. Int Microbiol (2010) 13(2):59–65. 20890840 10.2436/20.1501.01.111

[99] Brogden KA Polymicrobial Diseases of Animals and Humans. Washington DC: ASM Press (2002). 21735561

